# Altered Amygdala Development and Fear Processing in Prematurely Born Infants

**DOI:** 10.3389/fnana.2016.00055

**Published:** 2016-05-18

**Authors:** Anca Liliana Cismaru, Laura Gui, Lana Vasung, Fleur Lejeune, Koviljka Barisnikov, Anita Truttmann, Cristina Borradori Tolsa, Petra S. Hüppi

**Affiliations:** ^1^Division of Development and Growth, Department of Pediatrics, Hospital of GenevaGeneva, Switzerland; ^2^Child Clinical Neuropsychology Unit, University of GenevaGeneva, Switzerland; ^3^Division of Neonatology, University Hospital of LausanneLausanne, Switzerland

**Keywords:** amygdala, premature birth, fear, lab-TAB, amygdala development

## Abstract

**Context**: Prematurely born children have a high risk of developmental and behavioral disabilities. Cerebral abnormalities at term age have been clearly linked with later behavior alterations, but existing studies did not focus on the amygdala. Moreover, studies of early amygdala development after premature birth in humans are scarce.

**Objective**: To compare amygdala volumes in very preterm infants at term equivalent age (TEA) and term born infants, and to relate premature infants’ amygdala volumes with their performance on the Laboratory Temperament Assessment Battery (Lab-TAB) fear episode at 12 months.

**Participants**: Eighty one infants born between 2008 and 2014 at the University Hospitals of Geneva and Lausanne, taking part in longitudinal and functional imaging studies, who had undergone a magnetic resonance imaging (MRI) scan at TEA enabling manual amygdala delineation.

**Outcomes**: Amygdala volumes assessed by manual segmentation of MRI scans; volumes of cortical and subcortical gray matter, white matter and cerebrospinal fluid (CSF) automatically segmented in 66 infants; scores for the Lab-TAB fear episode for 42 premature infants at 12 months.

**Results**: Amygdala volumes were smaller in preterm infants at TEA than term infants (mean difference 138.03 mm^3^, *p* < 0.001), and overall right amygdala volumes were larger than left amygdala volumes (mean difference 36.88 mm^3^, *p* < 0.001). White matter volumes were significantly smaller (*p* < 0.001) and CSF volumes significantly larger (*p* < 0.001) in preterm than in term born infants, while cortical and subcortical gray matter volumes were not significantly different between groups. Amygdala volumes showed significant correlation with the intensity of the escape response to a fearsome toy (r_s_ = 0.38, *p* = 0.013), and were larger in infants showing an escape response compared to the infants showing no escape response (mean difference 120.97 mm^3^, *p* = 0.005). Amygdala volumes were not significantly correlated with the intensity of facial fear, distress vocalizations, bodily fear and positive motor activity in the fear episode.

**Conclusion**: Our results indicate that premature birth is associated with a reduction in amygdala volumes and white matter volumes at TEA, suggesting that altered amygdala development might be linked to alterations in white matter connectivity reported in premature infants. Moreover, our data suggests that such alterations might affect infants’ fear-processing capabilities.

## Introduction

Children born prematurely have a high risk of developmental and behavioral disabilities (Larroque et al., 2004; Delobel-Ayoub et al., 2006). These include learning disabilities, behavioral problems and socio-emotional problems (Bhutta et al., 2002; Johnson and Marlow, 2011; Treyvaud et al., 2012) and are associated with an altered development of various brain regions (Ment et al., 2009; Kwon et al., 2014)., The amygdala is a key part of the neural circuitry implicated in emotion processing and social development during early life (Bauman and Amaral, 2008). It is a bilateral structure located deep in the medial temporal lobes, whose strategic position, close to the temporal horn of lateral ventricles, enables the integration of limbic and neocortical information, but also makes it particularly vulnerable to injuries associated with premature birth (Rutherford, 2002).

The amygdala has been shown to play an important role in a wide range of behavioral and psychosocial processes. A patient who suffered a bilateral amygdala lesion during adolescence had deficits primarily related to fear processing, while her social behavior was unaffected (Adolphs et al., 1995, 1998). Most reproducibly activated by social stimuli, such as facial expressions depicting fear (Morris et al., 1996), the amygdala is also activated during nonsocial testing paradigms, such as fear conditioning (Büchel et al., 1998; LaBar et al., 1998; Cheng et al., 2003) and viewing of threatening and fearsome nonsocial stimuli (Hariri et al., 2003). Therefore, Bauman et al. (2004) suggested that the amygdala does not play an essential role in development of social behavior, but has a modulatory role achieved through emotion regulation (e.g., fear responses) that might be particularly important early on in development (Bauman et al., 2004; Leppänen and Nelson, 2012).

Studies on prenatal development and connectivity of the amygdala in humans are scarce (Nikolić and Kostović, 1986; Ulfig et al., 1998; Müller and O’Rahilly, 2006; Vasung et al., 2010b; ten Donkelaar, 2015). The literature regarding early development of emotion and social behavior in preterm born children is also limited. A few studies have found evidence that the socio-emotional development is different in premature children (Hughes et al., 2002; Spittle et al., 2009). When compared with term born peers, preterm born infants had higher emotional dysregulation (anger, frustration) scores at 12 months corrected age (Langerock et al., 2013), with similar tendencies at 42 months of age (Witt et al., 2014). Although many studies demonstrated a clear relationship between early cerebral abnormalities and behavior at term age (Clark et al., 1988; Anderson and Doyle, 2003; Aust et al., 2014), few explored the role of the amygdala in this interplay.

The present study aimed to compare volumetric measures of the amygdala in very preterm and term born children at term equivalent age (TEA), and to assess the relationship between amygdala volumes and performance on a fear processing task in prematurely born children at 12 months of age.

## Materials and Methods

### Participants

Among the infants born between 2008 and 2014 at the University Hospitals of Geneva (HUG) and Lausanne (CHUV), who participated in longitudinal and functional imaging studies, the 81 infants in our study were chosen as the ones who had undergone an magnetic resonance imaging (MRI) at TEA, and whose T2 images were of sufficient quality to enable the manual delineation of the amygdala. After receiving approval from the Research and Ethics Committees of the HUG and CHUV, informed parental consent was obtained for each infant included in the study. Infants were assigned to two groups based on their gestational age (GA) at birth (World Health Organization, 2012): 52 were born very prematurely (<29 gestational weeks) and 29 were born at term (>37 gestational weeks). An overall description of both groups is shown in Table 1. Birth weight *z*-scores were calculated for each child using German standard data (Voigt et al., 2006).

**Table 1:** **Group characteristics**.

	Preterm born infants (*n* = 52)	Term born infants (*n* = 29)	*p*-value^a^
Male, N (%)	26 (50)	16 (55.2)	0.66
GA at birth, mean (SD), weeks	27.16 (1.09)	39.72 (1.02)	<0.001
GA at MRI time, mean (SD), weeks	40.05 (0.91)	40.28 (1.29)	0.38
Birth weight, mean (SD), g	930.5 (204.3)	3289.3 (343.9)	<0.001
Birth weight *z*-score, mean (SD)^b^	−0.09 (0.72)	−0.37 (0.77)	0.11
IVH, N (%)	2 (3.8)	1 (3.4)	1.00
PVL, N (%)	2 (3.8)	0 (0)	0.54
BPD, N (%)	23 (44.2)	0 (0)	<0.001
PDA, N (%)	25 (48.1)	0 (0)	<0.001
NEC, N (%)	2 (3.8)	0 (0)	0.41
Antenatal steroids, N (%)	36 (69.2)	0 (0)	<0.001

*Abbreviations: IVH, intraventricular hemorrhage; PVL, periventricular leukomalacia assessed by MRI at TEA; BPD, broncho-pulmonary disease; PDA, patent ductus arteriosus; NEC, necrotizing enterocolitis; SD, standard deviation. ^a^Group characteristics were compared using independent sample t-tests for continuous variables, and Chi-square or Fisher’s exact test for categorical variables, as appropriate. ^b^Computed as (weight—M)/SD, where the mean weight M and standard deviation SD were obtained according to the infant’s sex and gestational age (Voigt et al., 2006)*.

### Methods

#### MRI Acquisition

Neonates underwent MRI scans at TEA at the Radiology Departments of the HUG and CHUV without sedation. Scans were performed on a 3.0-T Siemens Tim Trio scanner to obtain T2-weighted contiguous slices with 1.2 mm thickness (echo time (TE) = 150 ms, repetition time (TR) = 4600 ms, voxel dimensions = 0.8 × 0.8 × 1.2 mm^3^). Four prematurely born infants had evidence of brain injury at TEA. Two had periventricular leukomalacia (PVL grade II/III) and the other two had intraventricular hemorrhage (IVH).

#### Image Segmentation and Volume Measurements

**Figure 1:**
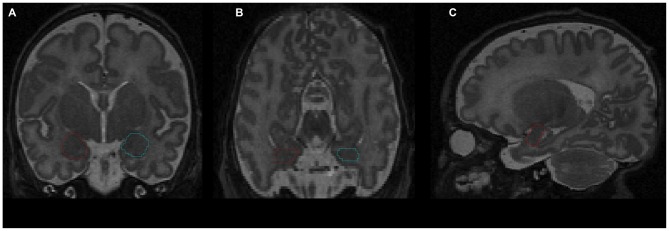
**Delineation of amygdala on T2-weighted magnetic resonance imaging (MRI) using the Amira software: (A) coronal view; (B) axial view; (C) sagittal view.** Red contour—right amygdala, blue contour—left amygdala.

The amygdala was segmented manually by a senior scientist with experience in the delineation of amygdala during human brain development (Vasung et al., 2008), who was blind to infants’ prematurity status. The segmentation was performed on T2-weighted MR cerebral images (Figure 1), using the manual contour editing function of a three dimensional (3D) visualization software (Amira). It was based on anatomical guidelines for the localization of the amygdala (Paxinos and Mai, 2004). Histological atlases were used for the validation and correction of anatomical landmarks (Bayer and Altman, 2003), the Allen Institute for Brain Science: http://www.brain-map.org/.

The amygdala is situated in the anteromedial temporal lobe, separated from the lentiform nuclei by a substantia inominata, and from the basal forebrain by a massive fiber system. It is a heterogeneous structure encompassing three parts: a specialized ventromedial expansion of the striatum, the caudal olfactory cortex, and a ventromedial extension of the claustrum (Swanson and Petrovich, 1998).

To delineate the amygdala, first the rostral pole of amygdala, easily recognizable by the appearance of the basolateral nuclei in Nissl stained sections (Schumann and Amaral, 2006), was defined as the most rostral part of the amygdala in sagittal MRI slices. Next, the amygdala was outlined on coronal slices using the following anatomical landmarks: the external capsule, anterior commissure, and temporal stem (dorsolateral border of amygdala), putamen and pallidum (superior border of amygdala), substantia innominata and nucleus basalis of Meynert (dorsomedial border of amygdala), semiannular sulcus (ventromedial border of amygdala, separating entorhinal cortex from the amygdala), temporal horn of the lateral ventricle and the hippocampus (caudal border of the amygdala), and white matter of temporal lobe (ventral border of the amygdala). After delineation of the amygdala on coronal slices, its borders were corrected in axial and sagittal slices. Once the outline was complete, the amygdala volume was calculated automatically and expressed in cubic millimeters.

Next, whole brain segmentation of the infants’ T2 MR scans was performed using a novel automatic method for newborn brain segmentation, based on knowledge of newborn brain morphology (tissue location, connectivity and structure; Gui et al., 2012). First the intracranial cavity (ICC) was segmented, and then, after masking out the cerebellum and brainstem, the following tissues were segmented: cortical and subcortical gray matter, white matter and cerebrospinal fluid (CSF). In some cases whole brain segmentation was not possible due to low image quality (movement artifacts), leaving a total of 66 (39 prematurely born and 27 term born) infants with available whole brain segmentations.

#### Fear-Processing Abilities Evaluated at 12 Months

The Lab-TAB test (Goldsmith and Rothbart, 1996) was designed to evaluate children’s temperament through a set of emotion-inducing behavioral episodes. Prematurely born infants who participated in longitudinal studies at the HUG and CHUV hospitals routinely undergo the Lab-TAB test. In our study of the amygdala, we were interested in the infants’ reaction to the fear episode of the Lab-TAB using the Unpredictable Mechanical Toy task, in which a mechanical robot dog walks towards the child. Once in front of the child, the dog stops and barks and howls before he moves back to the starting position. This procedure is designed to elicit fear and is split in three time intervals: (1) the dog approaching; (2) the first 5 s in front of the child; and (3) the next 5 s in front of the child. Each time interval is coded in accordance with the Lab-TAB manual for the following variables: intensity of facial fear, intensity of distress vocalizations, intensity of bodily fear, intensity of escape and startle response. Moreover, the variable “positive motor activity” was coded to account for all behaviors of pointing towards, reaching for or grabbing the mechanical toy. All variables were rated on 4-point Likert scales according to the intensity of a specific behavior (for example for the intensity of escape and startle: 0 = no escape; 1 = mild or fleeting escape; 2 = moderate escape, resulting in significant, but not extreme attempts to get away or resist; 3 = vigorous escape behavior).

All children were evaluated in a quiet room with at least one reference person present. Children were seated in front of a table, in a baby chair or on the reference person’s lap. All the evaluations were videotaped with parental consent for subsequent analysis.

Some infants did not want to perform or finish the cognitive testing, while others were excluded due to technical problems, weariness, stress or lack of interest, leaving a total of 42 prematurely born infants with Lab-TAB fear assessment at 12 months of age.

#### Statistical Analysis

Infants’ characteristics are reported according to the preterm/term group as mean ± standard deviation (SD) for continuous outcomes, and as frequency and percentages for categorical data. Characteristics’ differences between the two groups were assessed using a Student *t*-test for continuous variables, and using a chi-square test or Fisher exact test, as appropriate, for categorical variables.

Differences in amygdala volumes between preterm and term born groups, and between left and right hemispheres were assessed using a generalized linear model, with prematurity, gender and GA at the time of the MRI as between-subjects effects, and hemisphere side as within-subjects effect. Differences in brain tissue volumes between preterm and term born groups were assessed using general linear models, with prematurity, gender, GA at the time of the MRI and volume of ICC as between-subjects effects. For the sub-cohort for which the volumes of the ICC were available, differences in amygdala volumes between preterm and term born groups, and between left and right hemispheres were assessed using a generalized linear model, with prematurity, gender, ICC volume and GA at the time of the MRI as between-subjects effects, and hemisphere side as within-subjects effect.

The relationship between total amygdala volumes and infants’ response to the fear episode of the Lab-TAB at 12 months was assessed using Spearman correlations. Composite scores were binarized into equal to zero (no reaction to mechanical dog episode) and greater than zero (any reaction to mechanical dog episode), and amygdala volumes were compared between the reaction/no reaction infant groups with Student *t*-tests.

Statistical analysis was performed using SPSS Statistics Software. A two-tailed *p*-value ≤ 0.05 was considered significant for all analyses.

## Results

### Group Characteristics

Gender distribution, GA at the time of the MRI scan, birth weight *z*-scores and brain pathology distribution (IVH, PVL) were not significantly different between the preterm and the term born groups (Table 1). Morbidities associated with prematurity (broncho-pulmonary disease [BPD], patent ductus arteriosus [PDA] and necrotizing enterocolitis [NEC]) were not found in the term born group.

### Amygdala Volumes

Amygdala volumes (Tables 2, 3) were significantly smaller in preterm born infants at TEA than in term born infants, (mean difference 138.03 mm^3^, 95% confidence interval [CI], 108.62–167.45, *p* < 0.001), adjusting for gender and GA at MRI time. Moreover, volumes of right amygdala were significantly larger than volumes of left amygdala (mean difference 36.88 mm^3^, 95% CI, 23.63–50.12, *p* < 0.001), adjusting for gender and GA at MRI time. No significant relationship was found between amygdala volumes and gender or GA at MRI time. Amygdala volumes were not significantly different between the 5 infants presenting brain pathologies (IVH or PVL) and the other 76 infants of the cohort (Supplementary Table 1). Among the premature infants, amygdala volumes were not significantly different between infants presenting prematurity-associated morbidities (BPD, PDA, NEC) or having received antenatal steroids and the other premature infants of the cohort (Supplementary Table 1).

**Table 2:** **Amygdala volumes**.

	Preterm born infants (*n* = 52)	Term born infants (*n* = 29)
Left amygdala volume at TEA, mean (SD), mm^3^	592.78 (73.15)	723.54 (75.04)
Right amygdala volume at TEA, mean (SD), mm^3^	624.29 (73.46)	770.07 (71.95)

**Table 3:** **Parameter estimates for generalized linear model of amygdala volumes**.

	**B**	**95% CI**		*p*-value^a^
Preterm born	−138.03	−108.62	−167.45	<0.001
Right hemisphere	36.88	23.63	50.12	<0.001
Male	12.41	−16.09	40.91	0.39
GA at MRI	−1.83	−11.97	8.31	0.72

*^a^Generalized linear model with prematurity, gender and GA at the time of the MRI as between-subjects effects, and hemisphere side as within-subjects effect*.

### Whole Brain Segmentation Volumes

White matter volumes (Table 4, column of B values) were significantly smaller in preterm born infants at TEA compared to term born infants (mean difference 17.27 cm^3^, *p* < 0.001), when adjusted for gender, GA at the time of the MRI and volume of ICC. CSF volumes were significantly larger in preterm born infants at TEA compared to term born infants (mean difference 17.04 cm^3^, *p* < 0.001), when adjusted for gender, GA at the time of the MRI and volume of ICC. We did not find any significant differences in cortical gray matter and subcortical gray matter volumes between preterm and term born infants, when adjusted for gender, GA at the time of the MRI and volume of ICC (Table 4).

**Table 4:** **Whole brain segmentation volumes**.

Volume (cm^3^)	Preterm born infants (*n* = 39)	Term born infants (*n* = 27)	*p*-value^a^	B	95% CI	
CGM, mean (SD)	165.35 (18.13)	153.52 (17.58)	0.87	−0.46	5.88	4.95
SGM, mean (SD)	21.09 (1.68)	19.68 (2.05)	0.53	0.20	0.43	0.83
WM, mean (SD)	143.26 (14.48)	149.97 (16.64)	<0.001	−17.27	22.28	−12.25
CSF, mean (SD)	98.68 (23.00)	69.44 (19.98)	<0.001	17.04	−8.10	25.97

*CGM, cortical gray matter volume; SGM, subcortical gray matter volume; WM, white matter volume; CSF, cerebrospinal fluid volume. ^a^general linear models with prematurity, gender, GA at the time of the MRI and volume of ICC as between-subjects effects*.

Amygdala volumes (Table 5) were significantly smaller in preterm born infants at TEA than in term born infants (mean difference 165.64 mm^3^, 95% CI, 134.33–196.94, *p* < 0.001), adjusting for gender, ICC volume, and GA at MRI time. Moreover, volumes of right amygdala were significantly larger than volumes of left amygdala (mean difference 42.89 mm^3^, 95% CI, 28.48–57.30, *p* < 0.001) adjusting for gender, ICC volume, and GA at MRI time. No significant relationship was found between amygdala volumes and gender or GA at MRI time. Amygdala volumes were not significantly different between the 66 infants for whom whole brain segmentations were available and the other 15 infants of the cohort (Supplementary Table 1).

**Table 5:** **Parameter estimates for generalized linear model of amygdala volumes adjusting for ICC volume**.

	B	95% CI		*p*-value^a^
Preterm born	−165.64	−196.94	−134.33	<0.001
Right hemisphere	42.89	28.48	57.30	<0.001
Male	6.35	−24.42	37.13	0.69
GA at MRI	−9.33	−25.28	6.61	0.25
ICC volume	0.001	0	0.001	<0.001

*^a^Generalized linear model with prematurity, gender, ICC volume and GA at the time of the MRI as between-subjects effects, and hemisphere side as within-subjects effect*.

### Relationship Between Amygdala Volumes and Lab-TAB Fear Episode Scores

Composite scores for the five variables describing the infants’ reaction to the Unpredictable Mechanical Toy episode were computed by averaging over the three time intervals of the procedure (Larroque et al., 2004; Gagne et al., 2011; Table 6).

**Table 6:** **Lab-TAB fear composite scores (obtained by averaging scores for the three time intervals) in 42 prematurely born infants at 12 month of age**.

	Composite scores			
Variable	score = 0	0 < score ≤ 1	1 < score ≤ 2	2 < score ≤ 3
Facial fear, N (%)	15 (35.7)	18 (42.9)	7 (16.7)	2 (4.8)
Distress	37 (88.1)	4 (9.5)	1 (2.4)	0 (0)
vocalizations, N (%)
Bodily fear, N (%)	11 (26.2)	21 (50)	9 (21.4)	1 (2.4)
Escape response, N (%)	30 (71.4)	9 (21.4)	3 (7.1)	0 (0)
Positive motor activity, N (%)	16 (38.1)	12 (28.6)	11 (26.2)	3 (7.1)

The analysis of composite fear scores in the 42 prematurely born infants subjected to the fear episode of the Lab-TAB test showed significant positive correlation between the intensity of the escape response and total amygdala volumes (r_s_ = 0.38, *p* = 0.013). No significant correlation was found between total amygdala volumes and Lab-TAB scores measuring the intensity of facial fear, distress vocalizations, bodily fear and positive motor activity. Amygdala volumes were not significantly different between the infants who completed (*n* = 42) and those who did not complete (*n* = 10) the fear episode of the LabTAB test (Supplementary Table 1).

**Figure 2:**
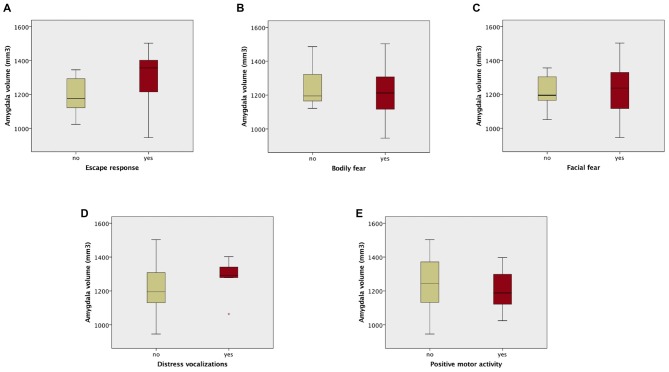
**Box plots of amygdala volumes dichotomized between infants who showed no fear reaction (composite score of corresponding variable = 0), and infants who showed any fear reaction (composite score of corresponding variable > 0) to the fearsome mechanical dog episode.** Reaction/no reaction infants groups were created based on the following composite scores: **(A)** escape response; **(B)** bodily fear; **(C)** facial fear; **(D)** distress vocalizations; **(E)** positive motor activity.

Amygdala volumes were significantly larger in infants showing an escape response compared to the infants showing no escape response (mean difference 120.97 mm^3^, Table 7, Figure 2). There were no significant differences in amygdala volumes between infants showing any reaction and infants showing no reaction of facial fear, bodily fear, distress vocalizations or positive motor action (Table 7, Figure 2).

**Table 7:** **Student *t*-test results comparing amygdala volumes between infants showing no reaction and infants showing any reaction to the mechanical dog episode (according to the corresponding composite score)**.

	Mean amygdala volume difference, mm^3^ (95% CI)	*t*	df	*p*-value
Facial fear	−1.87 (−87.75–84.01)	−0.044	40	0.965
Distress vocalizations	58.12 (−67.59–183.84)	0.934	40	0.356
Bodily fear	−36.30 (−129.18–56.58)	−0.790	40	0.434
Escape response	120.97 (38.48–203.45)	2.964	40	**0.005**
Positive motor activity	−43.55 (−127.14–40.05)	−1.053	40	0.299

## Discussion

### Amygdala Development and Preterm Birth

To our knowledge, this is the first quantitative MRI study of amygdala volumes (right, left and bilateral) in preterm infants at TEA and term-born infants. The study also explored the association between amygdala volumes at TEA and the response to a fear-eliciting situation in preterm infants at 12 months of age. Amygdala volumes in very preterm infants at TEA were significantly smaller than in term born controls, while a rightward hemispheric asymmetry was present in the whole cohort. Moreover, the evaluation of the fear response of preterm infants at 12 months of age revealed that the intensity of the escape response when faced with fear-eliciting stimuli was significantly correlated with amygdala volumes at TEA.

Our results can be interpreted in the light of the developmental path of the human amygdala. First discernable around 5 post conceptional weeks (Müller and O’Rahilly, 2006), the amygdala becomes an inhomogeneous structure around 12 post conceptional weeks (Nikolić and Kostović, 1986). From this point onward, it displays transient architectonic features, being composed of continuous cell columns separated by cell-sparse septa, which are linked to continuous neuronal migration and the presence of radial glial cells (Nikolić and Kostović, 1986; Ulfig et al., 2003). Most of amygdala’s neuronal cells come from the medial ganglionic eminence, with its medial nucleus developing slightly before the laterobasal one (Müller and O’Rahilly, 2006). While its basic cytoarchitecture is already defined at term (Humphrey, 1968; Ulfig et al., 1998), the amygdala still undergoes important structural and functional remodeling throughout its development from infancy to adolescence (Guo et al., 2007; Østby et al., 2009; Tottenham et al., 2009; Tottenham and Sheridan, 2010; Uematsu et al., 2012; Greimel et al., 2013). Many studies reported that the amygdala volumes tend to increase until early adolescence (Østby et al., 2009; Greimel et al., 2013), but the most rapid growth was shown to occur around birth (Vasung et al., 2008; Tottenham et al., 2009). Thus, our results showing lower amygdala volumes in preterm vs. term born infants at TEA reflect the vulnerability of the amygdala to premature birth and life ex-utero before term age, which seemingly cause disruptions of major developmental events taking place in the brain during the third trimester.

There are few quantitative MRI studies of regional brain volumes in preterm infants. Inder et al. (2005) reported lower cortical and subcortical gray matter volumes in preterm vs. term born infants at TEA, with major predictors of the altered volumes being GA at birth and the presence of white matter injury. Contrarily, Boardman et al. (2007) argued that brain tissue volumes of prematurely born infants at TEA are not reduced compared to term born infants, which is corroborated by our findings (Table 4). Rogers et al. (2014) did not find significant differences in the volumes of left or right amygdala between late preterm children (born between 34 and 36 gestational weeks) and term born peers at school age. In contrast to our study, these volumetric results were obtained at school age rather than term age, and only included late preterm children (compared to very preterm infants in our study), therefore the premature birth occurred in a period when brain development is more advanced, and thus less susceptible to being perturbed. In a study of preterm children from a wider range of GAs, Peterson et al. (2000) reported significantly smaller amygdala volumes in preterm children compared to term controls at 8 years corrected age. These results are in agreement with our study, suggesting that perturbations in amygdala development engendered by premature birth are apparent at TEA and may persist well into childhood.

Moreover, the fact that the volumetric differences between the groups in our study were still present when correcting for total intracranial volumes indicates that the reduction in amygdala volumes cannot simply be explained by head size differences between term and preterm infants. Peterson et al. (2000) came to similar conclusions in their study of 8 year old preterm infants.

While the current *in vivo* MRI-based study does not allow us to specify precisely what caused the volume reductions of the amygdala in the preterm infants, possible explanations could be found by looking at brain microstructure. Studies focusing on white matter fiber organization have found a reduction in brain microstructural maturity and connectivity in preterm children (Hüppi et al., 1998; Fischi-Gómez et al., 2014). Such findings indicate alterations of white matter development associated with prematurity, confirmed by our volumetric results (Table 4). Currently, it is not clear to which extent axonal fibers (composing white matter) contribute to the volume growth of amygdala during early development. However, taking into account that the peak of neuronal migration into amygdala occurs before premature birth, and our results indicate reduced white matter volumes in prematurely born infants (Table 4), we postulate that the smaller volumes of amygdala in prematurely born infants might reflect a decreased number of axonal connections of the amygdala, as well as alterations of the neurogenic processes influenced by axonal ingrowth (e.g., dendritic differentiation, cell growth; Vasung et al., 2008).

Further evidence supporting our hypothesis can be found by looking at amygdala connectivity findings. The first fibers of the amygdala are visible as early as 11 post conceptional weeks, while its abundant connectivity pattern can be seen by 17 post conceptional weeks (Vasung et al., 2010a). One of amygdala’s important connections involved in emotional significance processing and generation of emotional expressions (Schmahmann and Pandya, 2009) is the uncinate fascicle. The uncinate fascicle connects the anterior temporal lobe with the medial and orbital prefrontal cortex, and, as it passes through the temporal stem, it contributes some projections to the latero-basal amygdala. Prematurely born children were shown to have significantly lower fractional anisotropy (a measure of fiber tract organization) values of the uncinate fascicle at 12 years (Constable et al., 2008). This finding corroborates our hypothesis that such alterations in connectivity, also reflected by reduced white matter volumes in premature infants of our cohort, might affect the growth of the amygdala in preterm infants.

Our results also uncovered the presence of a rightward hemispheric asymmetry in amygdala volumes at TEA, demonstrating the early appearance of this asymmetry that was reported in adults by Uematsu et al. (2012). This is an important finding given the well-known functional hemispheric asymmetry present in adults, where direct electric stimulation of the right amygdala induces negative emotions, while the stimulation of the left amygdala induces both pleasant and unpleasant emotions (Lanteaume et al., 2007).

### Amygdala Development and the Regulation of Fear Responses

Research on prenatal development of the amygdala and the early development of socio-emotional competences is scarce. Studies on primates reported that subjects with neonatal amygdala lesions presented heightened fear responses when faced with social stimuli, and diminished fear responses when faced with (potentially threatening) non social objects (Prather et al., 2001; Bauman et al., 2004). However, when amygdala lesions were induced in adult primates, they showed no indication of social fear (Emery et al., 2001), but demonstrated similar diminished fear responses when faced with threatening objects (Kalin et al., 2001). Bauman et al. (2004) concluded that while the amygdala may not play an essential role in social behavior, it has a key contribution in the identification of potential dangers and the production of appropriate responses, thus indirectly modulating social behavior. These findings are in agreement with our results showing that premature infants with smaller amygdala volumes at TEA had a blunted reaction to a fearsome mechanical dog (non-social stimulus) at 12 months of age. These different fear-processing capabilities, seen as impaired identification of potential danger, might therefore be linked to the altered development of amygdala in prematurely born children. Moreover, our results are consistent with the conclusions of a behavioral study indicating that 12-month-old preterm infants displayed less fear reactivity than term born infants when confronted to the fearsome mechanical dog of the LabTAB test (Langerock et al., 2013). However, a limitation of our study was that fear processing capabilities could not be assessed in normally-developing term-born infants, since their parents considered extensive neuropsychological testing to be too time-consuming.

Literature concerning human adults with bilateral amygdala damage showed that such subjects were impaired in judging negative emotion in facial expressions and making accurate judgments of trustworthiness (Adolphs et al., 1995, 1998). Nevertheless, such findings cannot be extrapolated to the premature infant population, since socio-emotional competences undergo significant development during early childhood. Thus, the role of the amygdala in the development of socio-emotional difficulties in preterm infants needs to be evaluated in further studies focusing on amygdala connectivity and functional response.

## Conclusion

The present study brings new insights for understanding the impact of prematurity on brain development. Our data showed that amygdala volumes and whole brain white matter volumes where significantly smaller in preterm infants at TEA than in term born infants. Such findings reflect the altered growth of the amygdala associated with prematurity, which might be explained by modifications in white matter connectivity affecting the amygdala. Moreover, we presented evidence that a rightwards-hemispheric asymmetry of the amygdala is already present at TEA. In addition, in an exploratory analysis, we found that prematurely born children showing blunted escape responses to a fear-eliciting object had smaller amygdala volumes, suggesting that altered amygdala development might be linked with an impaired identification of fearsome and potentially dangerous objects in infancy. Further longitudinal studies of premature cohorts, as well as animal models of premature birth are needed to investigate the precise causes of such alterations in amygdala development associated with prematurity, as well as their long term developmental consequences. However, our preliminary findings have important clinical implications, as the detection of these regional abnormalities can improve early-targeted interventions.

## Author Contributions

ALC analyzed the data and wrote the manuscript. LG segmented the MRI images, helped with statistical analysis and manuscript revision. LV interpreted data, delineated amygdala, helped with statistical analysis, and wrote the manuscript. FL and KB evaluated prematurely born infants on fear episode of the LAB-TAB using the Unpredictable Mechanical Toy task at 12 month of age. AT was responsible for the MRI acquisition of infants at TEA. CBT designed the study and helped with interpretation. PSH designed the study, was responsible for the MRI acquisition of infants at TEA and helped with interpretation of the results.

## Funding

This work was supported by Swiss National Science Foundation, Project SPUM: from Cortex to Classroom: enhancing Brain Development for Premature Infants No. 140334, to PSH, and by the Swiss National Science Foundation grant 32473B_135817 “Development of emotion and cognition in preterm born cohorts: neurostructural and neurofunctional correlates from birth to early adolescence”.

## Conflict of Interest Statement

The authors declare that the research was conducted in the absence of any commercial or financial relationships that could be construed as a potential conflict of interest.
