# Supplementary material for: Altered Amygdala Development and Fear Processing in Prematurely Born Infants
Source: Front Neuroanat. 2016 May 18;10:55. doi: 10.3389/fnana.2016.00055 (PMC4870280; doi:10.3389/fnana.2016.00055)
Supplement: Supplementary file 1 [file Table_1.docx]

| Table 1. Student t-test results comparing total amygdala volumes (in mm^3^) between infants who had or not a brain pathology (IVH or PVL), premature infants who had or not BPD, PDA, NEC, or antenatal steroids, infants who had or not whole brain segmentation results, and premature infants who completed or not the fear episode of the Lab-TAB test. | | | | |
| --- | --- | --- | --- | --- |
|  | **Mean amygdala volume difference, mm^3^ (95% CI)** | **t** | **df** | **p-value** |
| Brain pathology (IVH, PVL), 5 out of 81 infants | -80.82 (-253.79, 92.14) | -0.930 | 79 | 0.36 |
| BPD, 23 out of 52 premature infants | 20.18 (-55.01, 95.36) | 0.539 | 50 | 0.59 |
| PDA, 25 out of 52 premature infants | -17.45 (-92.24, 57.34) | -0.469 | 50 | 0.64 |
| Antenatal steroids, 36 out of 52 premature infants | -66.08 (-145.02, 12.86) | -1.681 | 50 | 0.10 |
| NEC, 2 out of 52 premature infants | -97.34 (-290.10, 95.42) | -1.014 | 50 | 0.32 |
| Whole brain segmentation, 66 out of 81 infants | 63.66 (-16.49, 143.80) | 1.614 | 34.26^a^ | 0.12 |
| Fear episode, 42 out of 52 premature infants | 38.01 (-56.40, 132.41) | 0.809 | 50 | 0.42 |
| Abbreviations: IVH, intraventricular hemorrhage; PVL, periventricular leukomalacia assessed by MRI at TEA; BPD, broncho-pulmonary disease; PDA – patent ductus arteriosus; NEC – necrotizing enterocolitis;  ^a^ unequal variances between groups | | | | |
